# Supplementary material for: Topical ABT-263 treatment reduces aged skin senescence and improves subsequent wound healing
Source: Aging (Albany NY). 2024 Dec 3;17(1):16–32. doi: 10.18632/aging.206165 (PMC11810067; doi:10.18632/aging.206165)
Supplement: Supplementary Figures [file aging-17-206165-s001.pdf]

SUPPLEMENTARY FIGURES

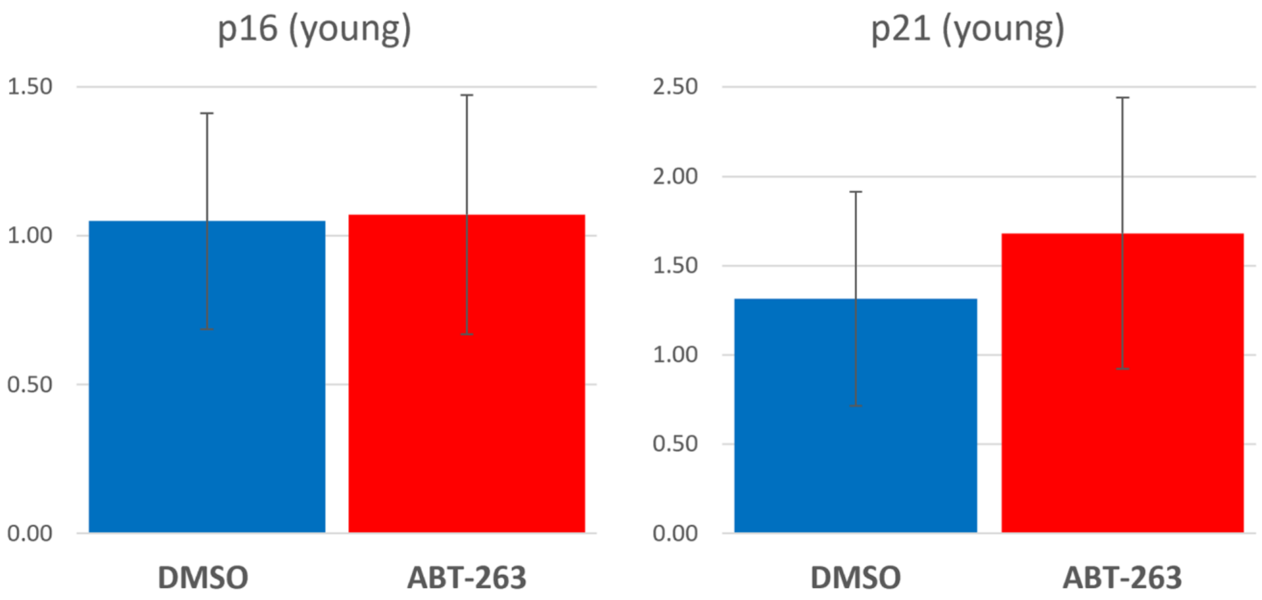

Supplementary Figure 1. Topical ABT-263 does not alter p16 or p21 expression in young mouse skin. p16 and p21 gene expression relative to β-actin after 5 days of ABT-263 (N=5) vs. DMSO (N=5), 2-month-old mice. t-test, * $p<0.05$.

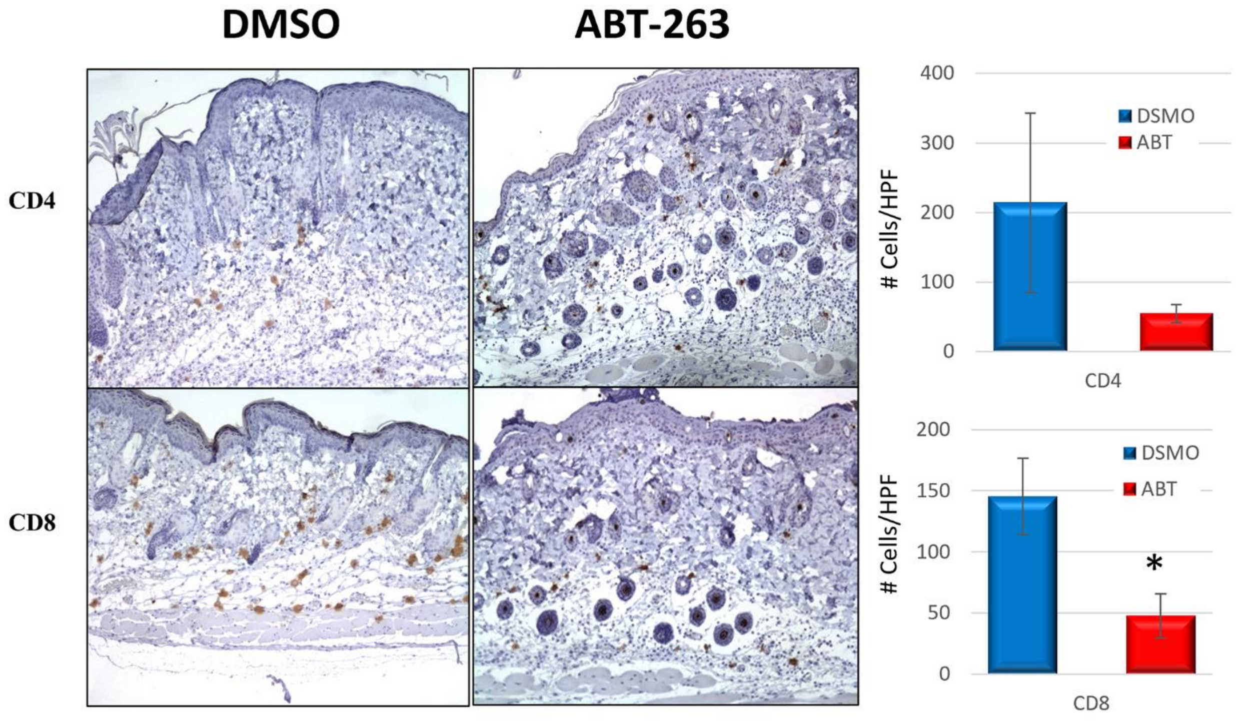

Supplementary Figure 2. Topical ABT-263 decreased CD4 and CD8 cell infiltration in aged skin. CD4 and CD8 staining of skin after 5 days of ABT-263 (N=5) vs. DMSO (N=5), 24-month-old mice. Number of cells/high-powered field. t-test, * $p<0.05$ significance level.

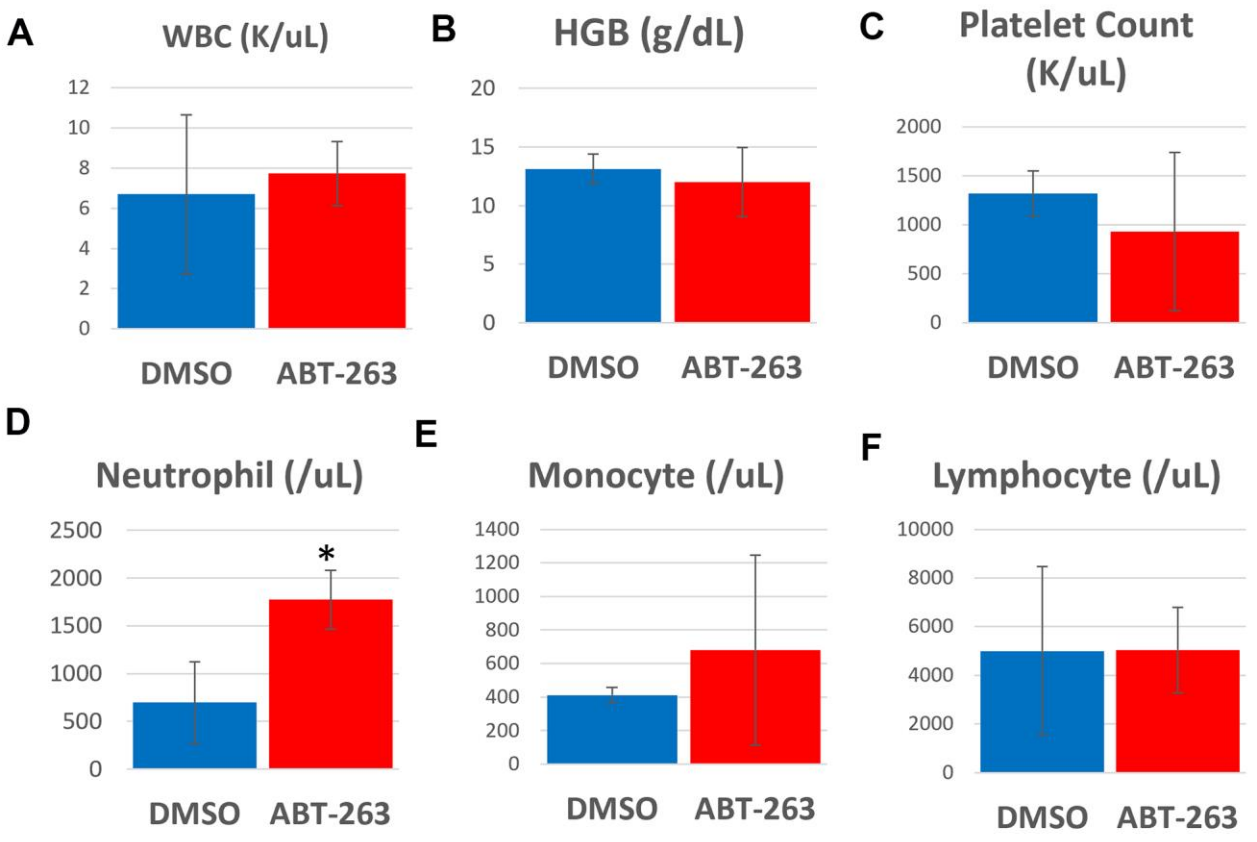

Supplementary Figure 3. Topical ABT-263 effects on systemic blood counts in aged mice. (**A**) White blood cell count. (**B**) Hemoglobin. (**C**) Platelet count. (**D**) Neutrophil count. (**E**) Monocyte count. (**F**) Lymphocyte count. Blood was obtained one day after 5 days of topical treatment with ABT-263 (N=3) vs. DMSO (N=2) in 24-month-old mice. t-test, * $p<0.05$ significance level.
